# Immune landscape in Burkitt lymphoma reveals M2-macrophage polarization and correlation between PD-L1 expression and non-canonical EBV latency program

**DOI:** 10.1186/s13027-020-00292-w

**Published:** 2020-05-06

**Authors:** Massimo Granai, Lucia Mundo, Ayse U. Akarca, Maria Chiara Siciliano, Hasan Rizvi, Virginia Mancini, Noel Onyango, Joshua Nyagol, Nicholas Othieno Abinya, Ibrahim Maha, Sandra Margielewska, Wenbin Wi, Michele Bibas, Pier Paolo Piccaluga, Leticia Quintanilla-Martinez, Falko Fend, Stefano Lazzi, Lorenzo Leoncini, Teresa Marafioti

**Affiliations:** 1 Department of Medical Biotechnology, University of Siena, Siena, Italy; 2 University Hospital of Tübingen, Institute of Pathology, Tübingen, Germany; 3 Department of Pathology, University College London, London, UK; 4 Department of Cellular Pathology, Barts Health NHS Trust, London, UK; 5 Department of Clinical Medicine and Therapeutics, University of Nairobi, Nairobi, Kenya; 6 Department of Human Pathology, University of Nairobi, Nairobi, Kenya; 7 South Egypt Cancer Institute, Assiut University, Assiut, Egypt; 8 Institute of Immunology and Immunotherapy, University of Birmingham, Birmingham, UK and Durham University, Durham, UK; 9 Clinical Department, National Institute for Infectious Diseases “Lazzaro Spallanzani” I.R.C.C.S, Rome, Italy; 10 Department of Experimental, Diagnostic, and Specialty Medicine Bologna University Medical School, S. Orsola Malpighi Hospital, Bologna and Euro-Mediterranean Institute of Science and Technology (IEMEST), Palermo, Italy; 11 Department of Cellular Pathology, University College Hospital, London, London UK

**Keywords:** Burkitt lymphoma, Tumour microenvironment, EBV, PD-L1, Immunotherapy, Immune checkpoint

## Abstract

**Background:**

The Tumor Microenviroment (TME) is a complex milieu that is increasingly recognized as a key factor in multiple stages of disease progression and responses to therapy as well as escape from immune surveillance. However, the precise contribution of specific immune effector and immune suppressor components of the TME in Burkitt lymphoma (BL) remains poorly understood.

**Methods:**

In this paper, we applied the computational algorithm CIBERSORT to Gene Expression Profiling (GEP) datasets of 40 BL samples to draw a map of immune and stromal components of TME. Furthermore, by multiple immunohistochemistry (IHC) and multispectral immunofluorescence (IF), we investigated the TME of additional series of 40 BL cases to evaluate the role of the Programmed Death-1 and Programmed Death Ligand-1 (PD-1/PD-L1) immune checkpoint axis.

**Results:**

Our results indicate that M2 polarized macrophages are the most prominent TME component in BL. In addition, we investigated the correlation between PD-L1 and latent membrane protein-2A (LMP2A) expression on tumour cells, highlighting a subgroup of BL cases characterized by a non-canonical latency program of EBV with an activated PD-L1 pathway.

**Conclusion:**

In conclusion, our study analysed the TME in BL and identified a tolerogenic immune signature highlighting new potential therapeutic targets.

## Background

Over recent years, the understanding of the biology of B-cell lymphomas has advanced significantly with the identification of the role played by the tumour microenvironment (TME) in lymphomagenesis [1, 2]. The TME of B-cell lymphomas mainly contains variable numbers of mesenchymal stem cells, immune cells and soluble factors. The complex interplay between tumour cells and TME regulates tumorigenesis and provides novel targets for immunotherapies [3, 4]. In aggressive lymphomas, particularly in BL, due to their high proliferation rate, intensive chemotherapy is required to counteract proliferation and dissemination of neoplastic cells. Unfortunately, these burdensome treatments are not as effective in elderly and immunocompromised patients [5]. Furthermore, in equatorial Africa, where BL is the most common childhood cancer, the prognosis of BL is still poor because the intensive therapeutic regimens often result in a severe neutropenia, with fatal consequences in resource poor settings [6–10]. Shortcomings of current BL therapies make the exploration of new therapeutic avenues a substantial and reasonable aim [7]. Therefore, a proper characterization of the TME in BL might be helpful to identify alternative therapeutic targets.

One of the histological hallmarks of BL is the high content of tumour-associated macrophages (TAMs) involved in apoptotic tumour cell clearance that confer the so-called starry-sky appearance [11]. Although little is known about the functional status of macrophages and their impact on tumour immune response in BL, TAMs may function as potential mediator of tumour progression through secretion of chemokines, cytokines and expression of immune checkpoint-associated proteins as PD-L1 [12, 13].

The expression of PD-L1 in B-cell lymphoma remains controversial, especially in BL. Indeed, PD-L1 has been reported in 80% of BL cases (8 out of 10) by Majzner [14]. However, this result was not reproduced by others [15]. Moreover, the role of the antigenic signature of Epstein Barr virus (EBV) in modulating the tumour microenvironment and the expression of immune-tolerant proteins has not been analysed in any of these studies. These different and somehow discordant results may be due to the diverse latency program of EBV infected cells and thus to different patterns of viral genes expression.

The constitutive association between EBV and BL, especially with endemic Burkitt lymphoma raises questions regarding the role of the virus in altering and actively shaping the tumour microenvironment [16–20]. Indeed, EBV orchestrates a variety of complex mechanism favouring the escape of lymphoma cells from anti-tumour immune responses while promoting the creation of niches in which tumour cells may find support for their growth and survival [19–22].

Computational methods such as GEP deconvolution allow high sensitivity discrimination of cell subsets within complex tissues, as tumours [23]. These approaches provide quantitative/ functional information also on rare tumour-infiltrating elements, offering the unprecedented opportunity of reanalysing available genomic data and identifying the immune signature. Here, we applied the computational algorithm CIBERSORT to GEP datasets of 40 BL samples previously published by our group [24], including endemic BL (eBL), sporadic BL (sBL) and immudeficiency associated BL (idBL) cases, to draw a map of immune and stromal components of TME. Finally, in order to validate GEP preliminary data, we applied multiplex immunohistochemistry to an additional cohort of 24 cases. These results were further supported by Vectra analysis of additional 16 BL by immunofluorescence. Thus, a total of 80 BL cases were included in the study.

In addition, we investigated the PD-1/PD-L1 pathway activation status and the contribution of EBV in PD-L1 induction as alternative mechanism responsible for immune evasion.

## Methods

### CIBERSORT and gene set enrichment analyses

A CIBERSORT-based deconvolution of GEP datasets (GSE26673) from 40 BL samples (13 eBLs, 21 sBLs, 6 idBLs; discovery cohort), previously published [24], was carried out using a 547-gene signature matrix customized for characterizing tissue sample immune cell composition, according to CIBERSORT instructions (https://cibersort.stanford.edu/) [23]. Briefly, normalized gene expression data were used to infer the relative proportions of 22 types of infiltrating immune cells while gene expression datasets were prepared using standard annotation files and data uploaded to the CIBERSORT web portal (http://cibersort.stanford.edu/), with the algorithm run using the default signature matrix at 1000 permutations. Gene set enrichment analysis (GSEA) was run on GSE26673.

### Multiplex immunohistochemistry

Multiplex immunohistochemistry was performed on 24 FFPE BL cases (12 eBL, 8 sBL and 4 idBL; validation cohort 1) which were retrieved from the Departments of Histopathology, University College Hospital, London (UK); Medical Biotechnologies, University of Siena, Siena (Italy); University of Nairobi, Nairobi (Kenya) and Istituto Lazzaro Spallanzani, Rome (Italy). The diagnosis of BL was issued by expert hematopathologists following the criteria described in the revised 4th edition of World Health Organization classification of tumours of Haematopoietic and Lymphoid Tissue [25]. Single immunohistochemistry for the diagnostic antibodies, for EBV antigens and EBV in situ hybridization was carried out on the Bond III Autostainer (Leica, Microsystems, Newcastle upon Tyne, UK) by following the manufacturer’s instructions.

By applying multiplex immunostaining (IHC), we investigated the simultaneous expression of: a) CD68 (Abcam, ab 955, 1:150) brown, CD-163 (Abcam, Ab87099, 1:100) red and C-Maf (Abcam, Ab243901, 1:150,) blue; b) PD-1 (Abcam, NAT105, 1:100) brown and CD8 (Leica Biosystem,4B1, 1:200) red and Granzyme B (Abcam134933, marker for T- cell activation) blue c) PD-L1 (ab238697, Abcam, 1:100) brown, CD-163 (Abcam, Ab87099, 1:100) red and MYC (Abcam, Ab32072, Y69 clone, 1:150) in blue;. The triple immunostaining was assessed as previously described [26]. The colour assignment and staining location are: a) PD-L1 brown/membranous; CD-163 red/membranous; C-MYC blue/nuclear b) PD-L1, brown/membranous; CD163 red/membranous; C-Maf blue/nuclear; c) PD-1 brown/membranous; CD8 red/membranous and Granzyme B blue/nuclear. Tissue sections from the same set of cases and without antibody/chromogens were used as negative control.

The percentage of each cell population characterized by multiplex immunostaining was calculated by counting the individual cell types in 10 hpf using a 40x objective (NIKON Eclipse E400).

### Multiplex immunofluorescence staining

Multiplex immunofluorescence (mIF) was carried out on 16 formalin fixed paraffin embedded (FFPE) endemic BL cases (validation cohort 2), belonging to set of samples previously studied and well characterized for EBV latency program [27].

Multiplex IF was applied to simultaneously detect the expression of: a) CD68 (Abcam, ab 955, 1:150) and CD163 (Leica Biosystem, 10D6, 1:200); b) PD-L1 (Dako, clone 22C3, 1:100) and CD163 (Leica Biosystem, 10D6, 1:200); c) PD-L1 and EBV-LMP2A (Abcam, clone 15F9, ab59028, 1:200). These double stainings use red and green or magenta and green chromogens. The colour assignment and staining location are: a) CD68 red/membranous; CD163 green/membranous; b) PD-L1, green/membranous and CD163, pink/membranous or PD-L1, green/membranous and CD163, red/membranous; c) PD-L1, red; LMP2A green/ membranous. The staining procedure was established according to previously published work [28]. Tissue sections from the same set of cases and without antibody/fluorophore were used as negative control. Multiplex IF staining reaction and image analysis (including quantification of antibodies expression) were performed using the Vectra 2.0 system (PerkinElmer, Waltham, MA) and Tissue FAXSFluo slide scanning system (TissueGnostics, Vienna Austria) based on a Zeiss Axio Imager Z2 upright epifluorescence microscope.

## Results

### EBV status

EBV status and the viral proteins expression in all the study cases are reported in Table 1. In particular, in the discovery cohort EBV was positive in all of the eBL, in 6 out of 21 sBL and in 5 out of 6 idBL.

**Table 1:** EBV status and the viral protein expression. Note: NA stands for not available

		EBER/EBNA1+	EBNA1+/LMP1+	EBNA1+/LMP2A+	EBNA1+/LMP1+/LMP2A+	EBV-	Total
Discovery cohort	**eBL**	13	NA	NA	NA	–	13
	**sBL**	6	NA	NA	NA	15	21
	**idBL**	5	NA	NA	NA	1	6
Validation cohort 1 (mIHC)	**eBL**	10	–	2	–	–	12
	**sBL**	2	–	–	–	6	8
	**idBL**	4	–	–	–	–	4
Validation cohort 2 (mIF)	**eBL**	11	1	4	–	–	16
		51	1	6	–	22	80

In the validation cohort 1, all of the eBL cases (12/12) were EBV positive, of which 10 expressed only EBNA1 by IHC while the remaining two were characterized by a non-canonical latency of EBV with the expression of both EBNA1 and LMP2A. Only two out of the 8 sBL cases were EBV positive showing a latency I expression pattern with the sole positivity of EBNA1. All of the idBL cases (4/4) were EBV positive and showed a latency of type I. In the validation cohort 2, all the eBL cases were EBV positive, with 11 out of 16 cases showing an EBV type I latency, while the remaining 5 cases exhibited a non-canonical EBV latency consisting of EBNA1 and LMP1 expression in one case and EBNA1 and LMP2A in the other 4.

### CIBERSORT identifies M2-polarized macrophages as the most representative TME component in BL

**Fig. 1:**
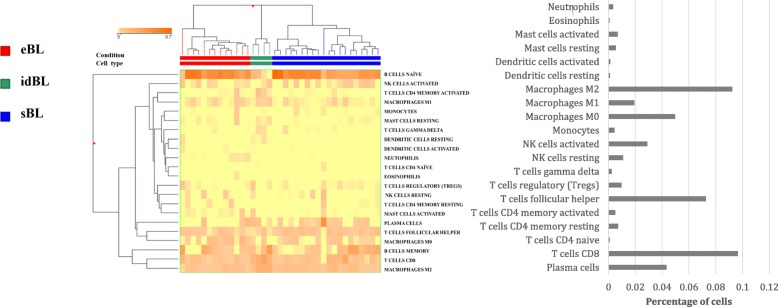
CIBERSORT algorithm. Analysis of 22 immune cell populations profiled by a previous GEP dataset (GSE26673) in BL tumor biopsies. The heat map figure has been generated by CIBERSORT webserver. The analysis showed that macrophages with a M2 profile were the most represented population followed by CD8-positive T-lymphocytes and CD4 follicular helper cells. In contrast, regulatory T-cells and M1 macrophages were poorly represented

The computational algorithm CIBERSORT to GEP datasets from BL samples revealed a heterogeneous reactive milieu with slight differences in tumour microenvironment among the three BL subtypes (eBL, sBL and idBL), most likely reflecting their underlying immunological status. The analysis showed that macrophages with a M2 profile were the most represented population followed by CD8-positive T-lymphocytes and CD4 follicular T-helper cells. In contrast, regulatory T-cells and M1 macrophages were poorly represented (Fig. 1).

### Multiplex IHC showed M2 macrophage polarization, cytotoxic T cells exhaustion and PD-L1 expression in Burkitt lymphoma cells

**Fig. 2:**
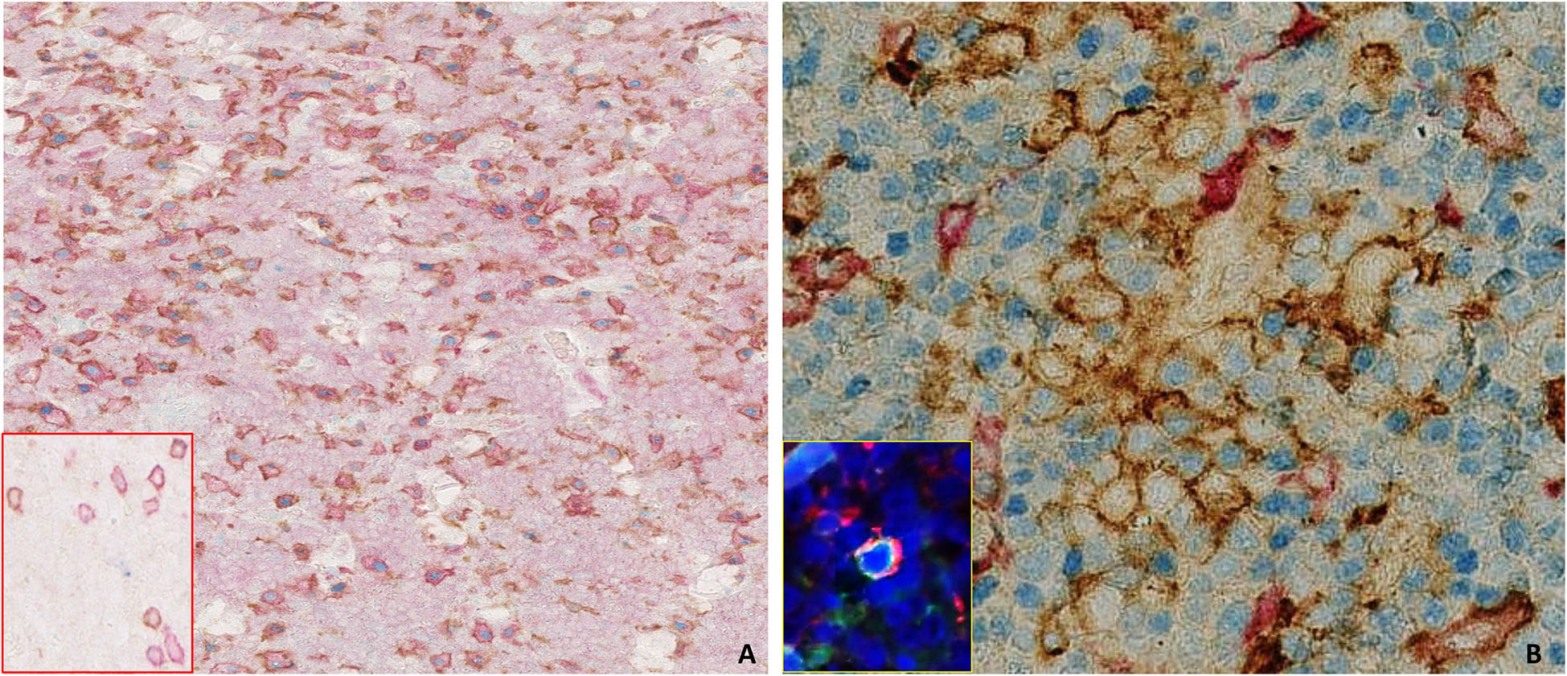
Macrophage polarization and PD-L1 expression on TAMs: (**a**) CD68 (brown), CD163 (red), c-maf (blue). The majority of TAMs express M2 phenotype markers (CD163+, c-maf+). (O.M: 10x). Inset: CD8 (red), Granzyme B (blue) and PD-1(brown), pattern of PD1 expression on cytotoxic T cells. (**b**) C-MYC (blue),PD-L1 (brown), CD163 (red); the majority of TAMs in eBL and idBL cases expressed PD-L1, in addition triple staining disclosed clusters of C-MYC/PD-L1 double positive cells in 2 cases characterized by co-expression of LMP2A (Inset double IF) clustering with TAMs (O.M: 40x)

The analysis confirmed a shift towards M2 phenotype (CD68 + CD163+/c-maf +) in all BL cases of validation cohort 1 ranging from 60 to 80% of total TAMs (Table 2, Fig. 2a). In particular, the evaluation of M1 macrophages, defined by CD68+, CD163-, c-maf- cells showed very similar values among the series ranging from 20 to 40% of total TAMs. CD8/ PD1/ Granzyme B staining highlighted that the vast majority of CD8+ T cells co-expressed PD-1 ranging from 60 to 80 and 50% to 70% of total tumor infiltrating cytotoxic T cells for eBL and idBL respectively (insert Fig. 2a; Table 2). Interestingly, sBL cases, in which EBV was negative to a greater extent (6 out of 8 cases) showed a markedly lower PD1 expression on CD8 positive T cells (from 35 to 50% of total CD8+ cells; Table 2).

**Table 2:** mIHC for TAMs and PD-L1 expression on 24 BL samples (validation cohort 2) stained for PD-L1, CD68, CD163 and c-maf; mIHC for cytoxic T cells on 24 BL samples (validation cohort 2) stained for PD1, CD8 and granzyme B

mIHC for PD-1/PD-L1 expression and macrophage polarization				
		eBL (*n* = 12)	sBL (*n* = 8)	iBL (*n* = 4)
TAM	M1 (CD68+/CD163-/c-maf−)	20–40%	30–40%	20–30%
	M2 (CD68+/CD163+/c-maf+)	60–80%	60–70%	70–80%
PD-L1	TAMs (PD-L1+/CD163+)	65–80%	20–40%	55–75%
	BL cells (MYC+/PD-L1+)	10–30%	0–10%	0–10%
Exhausted cytotoxic T cells	CD8+/PD1+/granzyme B-	60–80%	20–40%	60–80%

The vast majority of TAMs in eBL and idBL cases expressed PD-L1 ranging from 65 to 80% and from 55 to 75% (Fig. 2b, Table 2). Interestingly, PD-L1 expression on TAMs in sBL showed lower values (from 20 to 40%; Table 2). In addition, C-Myc/PD- L1/CD163 triple staining also disclosed clusters of MYC/PD-L1 double positive cells in 2 eBL cases characterized by expression of LMP2A. PD-L1 expression in these cases was focal and heterogeneous with a degree of intensity from weak to strong in 10–30% of the total tumor cells, clustering with TAMs (Fig. 2b). The co-expression of PD-L1 and LMP2A in scattered neoplastic cells was then confirmed by double IF (insert Fig. 2b), identifying a possible correlation between EBV and LMP2A in PD-L1 induction. Of note, EBV negative BL (6 out of 24 cases) and conventional BL cases with canonical EBV type I latency characterized by the sole expression of EBNA1 (16 out of 24 cases) showed low or absent PD-L1 positivity ranging from 0 to 10% of total tumor cells. These findings indicate that PD-L1 checkpoint activation is more likely related to an unusual latency program of EBV rather than to the EBV presence itself.

### Multiplex immunofluorescence confirmed the prevalence of M2 macrophages and revealed a heterogeneous PD-L1 expression

Tissue samples were studied by mIF and analysed by VECTRA to quantify macrophages and PD-L1 expression on 16 BL samples (validation cohort 2) stained for PD-L1, CD68 and CD163.

**Fig. 3:**
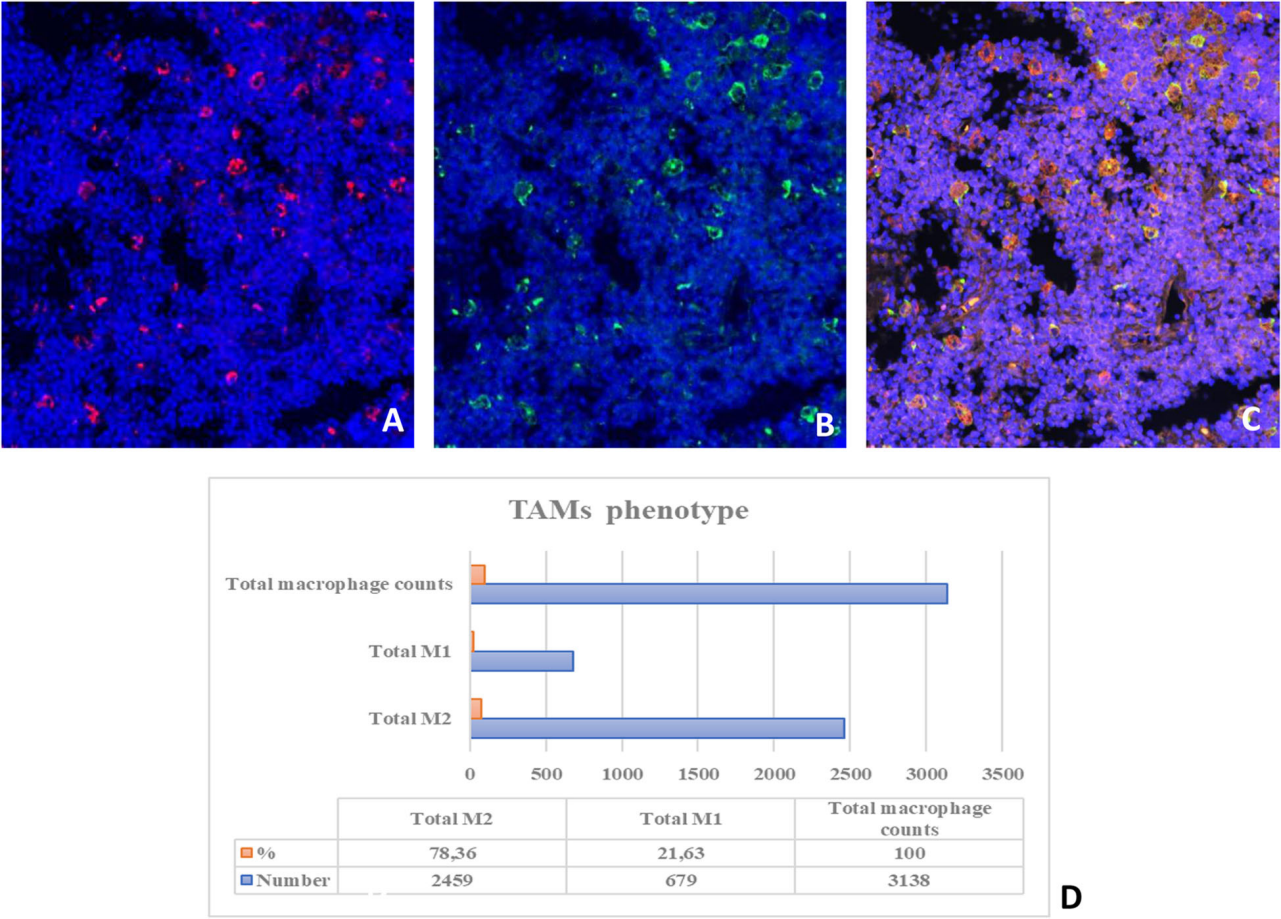
Immunofluorescence staining for Tumor-associated Macrophage Polarization in BL. (**a**) CD68 (red) (**b**) CD163 (green). Nuclei were stained with DAPI. **c** shows merge of A,B pictures. (**d**) Example of total macrophage count by Vectra analysis in one case

**Fig. 4:**
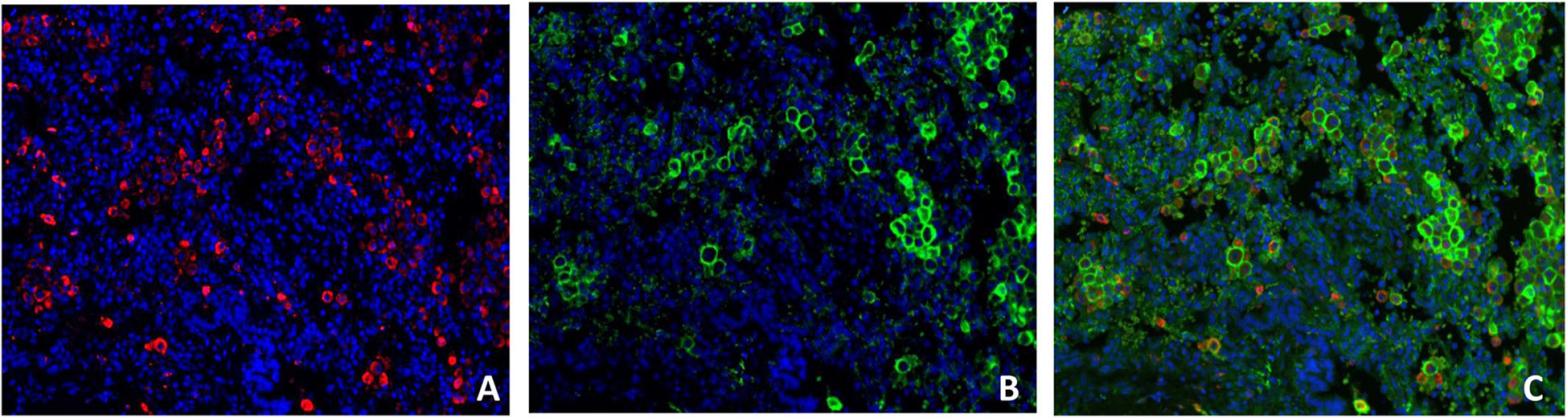
Immunofluorescence staining for PD-L1 and TAMs. **a** CD163 (red), **b** PDL-1 (green) merge (**c**). The vast majority of TAMs exhibited M2 phenotype markers (CD163+) and expressed PD-L1 to a greater extent

In all the cases in validation cohort 2 the M2 macrophages were the most represented population ranging from 66 to 78% of total TAMs (Fig. 3), thus confirming the CIBERSORT and mIHC results. In addition, the vast majority of them were positive for PD-L1 with a range of expression from 35 to 70% of total macrophages (Fig. 4; Table 3).

**Table 3:** mIF and VECTRA analysis of macrophages and PD-L1 expression on 16 BL samples (validation cohort 2) stained for PD-L1, CD68 and CD163

mIF	
Macrophage polarization and PD-L1 expression	n
M1	22–34%
M2	66–78%
n (%) PDL1 (+) CD163(+)	35–70%

## Discussion

Although multiple studies have investigated TME and PD-L1 expression in B-cell lymphomas, only limited, small studies have been conducted in BL [14–16].

In the present study, we extensively evaluated the TME composition, activation status and expression of inhibitory immune checkpoints both on the inflammatory infiltrate and neoplastic cells of BL tumors including eBL, sBL and idBL cases. Thus, we investigated PD-L1 expression and the contribution of EBV in fostering the activation of the PD1-PD-L1 axis. The influence of the microenvironment on cell proliferation and destruction varies greatly according to the inherent histotype of the lymphoma cell type [29–31]. In particular, Hodgkin lymphoma (HL) tissue often consists of relatively few monoclonal cancer cells but at least 90% non-malignant cells (e.g., regulatory T cells), contributing to a rather unique surrounding immune ecosystem. On the other hand, BL seems to be largely devoid of such a supportive cellular environment, although the high content of Tumor-Associated Macrophages (TAMs) might play a distinct, specific, important role in neoplastic progression through secretion of chemokines, cytokines and immune checkpoint-associated proteins as PD-L1 [12, 13].

In the recent decade a model has been developed to describe the complex mechanism of macrophage activation as a polarization towards two opposite states, namely M1 and M2, with pro-inflammatory and pro-tumoral properties respectively [32–36]. TAM density in particular M2-TAMs have been associated with tumor progression and poor prognosis in DLBCL [37, 38].

In the present work, we identified a polarization towards a M2 phenotype of TAMs in all cases by applying three different approaches (CIBERSORT, mIHC and mIF) regardless of the subtype of BL, and thus, of EBV status.

These cells, intimately associated with the neoplastic cells, constituted also the major source of PD- L1, which may inhibit the overall inflammatory response and allow the neoplastic cells to evade antitumor immunity. However, the lower rate of PD-L1 expression on TAMs in sBL, as compared with eBL and idBL, which are frequently associated with EBV, may suggest a role of the virus in inducing PDL1 expression.

PD-L1 is a major regulator of T cell function and, after engaging PD-1, leads to an altered functional state of T-cells, namely T cell exhaustion [39]. In this regard, we found that in eBL and idBL the vast majority of the CD8+ infiltrating T cells expressed PD-1 highlighting an adaptive immune response resistance mechanism in such cases.

However, PD-L1 limits an antitumor immune response by signalling not only through PD-1 but also with nother receptor, namely CD80 (also named B7–1), expressed on the surface of activated CD8+ T cells [40–42]. The influence of the PD-L1/PD-1 interaction on CD8+ T cell function has been extensively characterized and is known to limit CD8+ T cell responses by inhibiting TCR signalling, thus restricting CD8+ T cell survival, proliferation, and cytokine production. On the other hand, the role of the PD-L1/CD80 pathway on CD8+ T cell functions in BL is unknown, and thus, further studies are necessary.

In addition, the role of PD-1 expression in Tumor Infiltrating Lymphocytes (TILs) on both lymphoid and epithelial malignancies is controversial [38]. PD-1 expression in CD8+ cells has been associated with the selective suppression of cytotoxic lymphocytes in EBV positive nasopharyngeal carcinoma [43]. On the other hand, the PD-1+ TILs have also been described to lack Tim-3 expression in papilloma virus positive cancers, and thus possibly representing activated T-cells [44].

Emerging evidence in EBV-related malignancies indicates that the virus possesses the ability to actively shape the tumor microenvironment, and favours its escape from anti-tumor immune responses through a variety of complex mechanisms [18]. EBV may induce a strong up-regulation of PD-L1 expression both directly on the surface of human primary monocytes, or indirectly on neoplastic cells, through its viral proteins LMP-1 which interfere with downstream cellular signalling (i.e. AP1; JAK/STAT) [45, 46] to induce an immune tolerant niche for EBV- related tumors [47–49]. LMP-2 may also exert its tolerogenic effect by affecting crucial cell-cycle regulating pathways such as PI3K/Akt wich plays a critical role in PD-L1 expression [50–52].

Although PD-L1 expression has been largely investigated in B cell lymphomas, the distinction of its expression in cellular microenviroment and/or in tumour cells has not been made in most studies [1–4].

Here we showed that EBV in BL might induce PD-L1 expression on tumor cells in a minority of cases characterized by a non-canonical latency with LMP2A positivity. On the other hand, it might influence PD-L1 upregulation on TAMs also in cases with canonical EBV latency I. However, the prevalence of M2 macrophages as primary constituent of the TME in BL is a constant finding in all BL subtypes and thus, macrophage polarization towards a pro-tumoral state seems an event related to the intrinsic characteristics of the tumor.

## Conclusions

In conclusion, although based on a small sample size, our findings may provide insights on BL TME and its underlying mechanisms of immune evasion. The cross-talk between different actors including TAMs, PD-1/PD-L1, T-cells, viral antigens and tumor cells may result in the failure of innate immunity in BL which results in M2 polarization. Despite the good response to conventional therapy of BL, our data may provide a rationale for new immunotherapeutic strategies.

## Data Availability

All data are available from the corresponding author.

## Abbreviations

TME: Tumor Microenviroment
BL: Burkitt lymphoma
GEP: Gene expression profiling
IHC: immunohistochemistry
IF: immunofluorescence
PD-1: Programmed Death-1
PD-L1: Programmed Death Ligand-1
LMP2A: Latent membrane protein 2A
LMP1: latent membrane protein 1
EBV: Epstein-Barr virus
TAM: Tumour associated macrophage
eBL: endemic Burkitt lymphoma
sBL: sporadic Burkitt lymphoma
idBL: immune deficiency Bukirtt lymphoma
GSEA: Gene set enrichment analysis
FFPE: formalin fixed paraffin embedded
HL: Hodgkin lymphoma
DLBCL: Diffuse large B cell lymphoma
