# Correction to: Immune landscape in Burkitt lymphoma reveals M2-macrophage polarization and correlation between PD-L1 expression and non-canonical EBV latency program

**DOI:** 10.1186/s13027-020-00304-9

**Published:** 2020-06-10

**Authors:** Massimo Granai, Lucia Mundo, Ayse U. Akarca, Maria Chiara Siciliano, Hasan Rizvi, Virginia Mancini, Noel Onyango, Joshua Nyagol, Nicholas Othieno Abinya, Ibrahim Maha, Sandra Margielewska, Wenbin Wei, Michele Bibas, Pier Paolo Piccaluga, Leticia Quintanilla-Martinez, Falko Fend, Stefano Lazzi, Lorenzo Leoncini, Teresa Marafioti

**Affiliations:** 1 Department of Medical Biotechnology, University of Siena, Siena, Italy; 2 Institute of Pathology, University Hospital of Tübingen, Tübingen, Germany; 3 Department of Pathology, University College London, London, UK; 4 Department of Cellular Pathology, Barts Health NHS Trust, London, UK; 5 Department of Clinical Medicine and Therapeutics, University of Nairobi, Nairobi, Kenya; 6 Department of Human Pathology, University of Nairobi, Nairobi, Kenya; 7 South Egypt Cancer Institute, Assiut University, Assiut, Egypt; 8 Institute of Immunology and Immunotherapy, University of Birmingham, Birmingham, UK; 9 Department of Biosciences, Durham University, Durham, UK; 10 Clinical Department, National Institute for Infectious Diseases “Lazzaro Spallanzani” I.R.C.C.S, Rome, Italy; 11 Department of Experimental Diagnostic, and Specialty Medicine Bologna University Medical School, Bologna; Euro-Mediterranean Institute of Science and Technology (IEMEST), Palermo, Italy; 12 Department of Pathology, JKUAT, Nairobi, Kenya; 13 Department of Cellular Pathology, University College Hospital, London, UK

**Correction to: Infect Agents Cancer (2020) 15:28**

**https://doi.org/10.1186/s13027-020-00292-w**

Following publication of the original article [1], the authors identified an error in the author name of Wenbin Wei

- The incorrect author name is: Wenbin Wi
- The correct author name is: Wenbin Wei

The author group has been updated above and the original article [1] has been corrected.
